# C-Reactive Protein Levels and Risk of Cardiovascular Diseases: A Two-Sample Bidirectional Mendelian Randomization Study

**DOI:** 10.3390/ijms24119129

**Published:** 2023-05-23

**Authors:** Annapurna Kuppa, Himi Tripathi, Ahmed Al-Darraji, Wadea M. Tarhuni, Ahmed Abdel-Latif

**Affiliations:** 1 Division of Cardiovascular Medicine, Department of Internal Medicine, University of Michigan, Ann Arbor, MI 48109, USA; 2 Canadian Cardiac Research Center, Department of Internal Medicine, Division of Cardiology, University of Saskatchewan, Saskatoon, SK S7N 5A2, Canada; wtarhuni@cardiaccentre.ca; 3 Ann Arbor VA Healthcare System, Ann Arbor, MI 48109, USA

**Keywords:** Mendelian randomization, sensitivity analysis, multivariable MR analysis, C-reactive protein, cardiovascular diseases, hypertensive heart disease, bidirectional Mendelian randomization analysis, two-sample Mendelian randomization, MR-PRESSO, PhenoScanner

## Abstract

Elevated C-reactive protein (CRP) levels are an indicator of inflammation, a major risk factor for cardiovascular disease (CVD). However, this potential association in observational studies remains inconclusive. We performed a two-sample bidirectional Mendelian randomization (MR) study using publicly available GWAS summary statistics to evaluate the relationship between CRP and CVD. Instrumental variables (IVs) were carefully selected, and multiple approaches were used to make robust conclusions. Horizontal pleiotropy and heterogeneity were evaluated using the MR-Egger intercept and Cochran’s Q-test. The strength of the IVs was determined using F-statistics. The causal effect of CRP on the risk of hypertensive heart disease (HHD) was statistically significant, but we did not observe a significant causal relationship between CRP and the risk of myocardial infarction, coronary artery disease, heart failure, or atherosclerosis. Our primary analyses, after performing outlier correction using MR-PRESSO and the Multivariable MR method, revealed that IVs that increased CRP levels also increased the HHD risk. However, after excluding outlier IVs identified using PhenoScanner, the initial MR results were altered, but the sensitivity analyses remained congruent with the results from the primary analyses. We found no evidence of reverse causation between CVD and CRP. Our findings warrant updated MR studies to confirm the role of CRP as a clinical biomarker for HHD.

## 1. Introduction

Cardiovascular diseases (CVDs) are the primary cause of death in the United States, prompting an urgent quest for novel and specific diagnostic and treatment options. CVD is a primary public health concern with an annual burden of more than USD 200 billion [1], an estimate that is expected to increase exponentially by 2060 [2]. CVDs are conditions that alter the structure and function of the heart, such as hypertensive heart disease (HHD), myocardial infarction (MI), heart failure (HF), coronary artery disease (CAD), and atherosclerosis. HF is defined as the incompetence of the heart muscle to supply the necessary amounts of oxygen and blood to meet the metabolic demands of the peripheral tissues [3]. Hypertensive heart disease (HHD) is a major public health concern, affecting millions of people worldwide. It is a complex condition characterized by the presence of hypertension and its impact on the heart’s structure and function. The etiology of HHD is multifactorial, involving genetic, environmental, and lifestyle factors that contribute to the development and progression of the disease [4]. Hypertension can lead to structural and functional changes in the heart, such as left ventricular hypertrophy, myocardial fibrosis, and diastolic dysfunction, which can ultimately result in heart failure [4]. The exact mechanisms underlying the development of HHD are not fully understood, but it is believed that increased wall stress, neurohormonal activation, and inflammation play crucial roles in the pathophysiology of the disease [5]. Lipids, T-cells, immune cells, fibrous elements, and macrophage accumulation in the innermost layer of the artery characterize atherosclerosis. The process of fibrous and fat accumulation leads to plaque formation. This accumulation is activated by inflammatory pathways and vessel narrowing, preceded by the activation of vascular endothelium. With time, atherosclerotic plaques accumulate more fibrous materials and undergo calcification [6]. The deposition of atherosclerotic plaques in the coronary arteries is the primary cause of CAD [7]. The rupture of preexisting plaques can lead to coronary artery thrombus, resulting in complete occlusion of the artery, which manifests clinically as MI [8]. While the etiology of CVD is not fully understood, inflammation, among other comorbidities/conditions such as type 2 diabetes, lifestyle, hypertension, obesity, chronic kidney disease, renal failure, and genetics, are some of the well-known CVD risk factors [9,10,11,12].

Inflammation promotes damage to endothelial cells and the progression of atherosclerotic plaques and, thus, plays a vital role in the progression and pathogenesis of CVD [13]. In the setting of MI, inflammation can be both valuable and detrimental as it is involved in infarct resolution, repair, and remodeling, but prolonged inflammation can also increase infarct size [14] and hamper cardiac recovery [15]. A large body of evidence from well-conducted human and animal studies suggests a foundational role for inflammation in the development of hypertension and vascular diseases [16,17,18,19]. C-reactive protein (CRP) is a routinely measured stable protein, a pro-inflammatory circulating cytokine, and a widely accepted indicator of systemic inflammation [13]. It is found in the plasma, where its concentrations increase in response to an injury, infection, or inflammation [20]. CRP binding to the complement system leads to a rise in apoptotic cells, components of the extracellular matrix, cell receptors, growth factors, and infarct size, which in turn is conducive to the progression of CVD [21].

Epidemiological and prospective cohort studies have independently reported an association between CRP levels and the risk of stroke, peripheral artery disease, acute myocardial infarction, ischemia, atherogenesis, and sudden cardiac death [16,22,23,24,25]. However, other recent genetic studies have failed to establish a causal relationship between CRP levels and the risk of CVD [23,24,26,27,28,29,30]. These conflicting results demand a careful exploration of the association between CRP and CVD risk. 

The Mendelian randomization (MR) approach is used to study the causal relationship between genetic variants, as instrumental variables, in the exposure and the risk of outcome [31]. This method considers any biases arising from confounding or reverse association compared to observational studies [31]. The genetic determinants regulate circulating levels of CRP at the *CRP* locus; however, these variants only account for a fraction of the variability. Including other powerful genetic determinants affecting the serum variability of CRP levels would help achieve more robust and reliable results. Therefore, we performed a two-sample MR study to investigate the relationship between single nucleotide polymorphisms (SNPs) associated with CRP levels and the risk of different CVD outcomes. We also performed a reverse MR analysis to study the effect of causality between CVD and CRP. We used publicly available GWAS summary statistics from UK Biobank, EBI GWAS, and FinnGen within European ancestry to perform our analysis. We also evaluated the robustness of our MR approach using multiple methods, such as univariable and multivariable MR accompanied by sensitivity analyses.

## 2. Results

**Figure 1:**
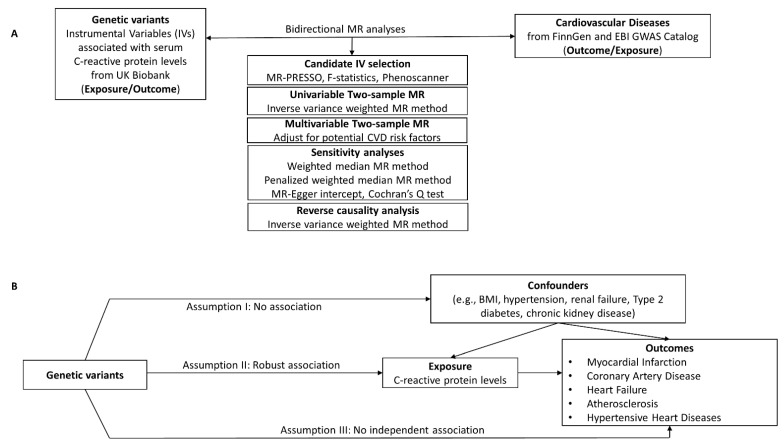
(**A**) Study design for the CRP-CVD two-sample bidirectional MR analyses. (**B**) The validity of the MR method depends on the instrumental variables (IVs) being satisfied for three key assumptions. Genetic instrumental variables were obtained for C-reactive protein levels from UK Biobank. GWAS summary statistics for CVD were obtained from FinnGen or EBI GWAS catalog. Two-sample MR analyses were performed for each exposure–outcome pair.

**Figure 2:**
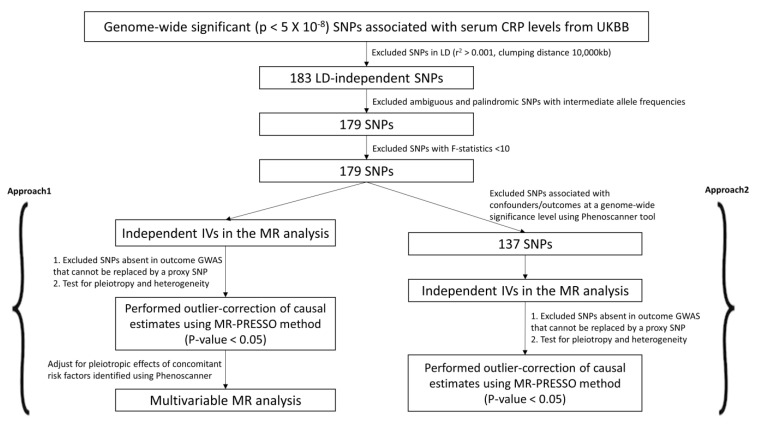
Candidate IV selection using quality control steps. We used two different approaches (Approach 1 and 2) to perform the two-sample MR study.

Previous evidence on the association between C-reactive protein (CRP) and cardiovascular disease (CVD) remains ambiguous. Therefore, we used two approaches (Approach 1 and 2) to select candidate IVs and study the causality between CRP and CVD (Figure 1 and Figure 2). We retrieved 183 LD-independent genome-wide SNPs associated with serum CRP levels. We excluded four SNPs (rs10203386, rs10810455, rs2057069, rs67581262) for being palindromic with intermediate allele frequencies to remove any potential biases. All 179 variants were robustly associated with the exposure at F-statistics > 10 (Table 1).

**Figure 3:**
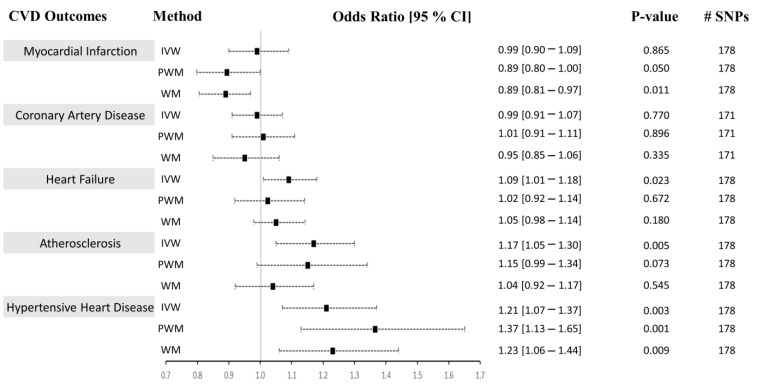
Causal associations between CRP levels and CVD using Approach 1. MR estimations were performed using IVW as the primary method and PWM and WM as sensitivity analyses. The effect sizes are shown by a black square with 95% confidence intervals as black dashed lines. The number of SNPs found in the outcome GWAS is shown in the #SNPs column. Abbreviations: cardiovascular diseases: CVD, inverse-variance weighted: IVW, weighted median: WM, penalized weighted median: PWM, 95% confidence interval: 95% CI.

In Approach 1, we independently used univariable and sensitivity methods (inverse-variance weighted (IVW), weighted median (WM), penalized weighted median (PWM), and MR-Egger method) in the two-sample Mendelian randomization (MR) analysis to determine whether CRP causally influences the risk of CVD (Figure 3). The characteristics of the candidate SNPs predictive of CRP are presented in Table 1. The 179 CRP-associated variants were consolidated and used as combined instrumental variables in the MR analysis (F-statistics_combined_ = 183.29). The number of SNPs found in the MR analysis for each outcome is listed in Figure 3. We present the univariable MR results using the IVM, WM, and PWM methods between CRP levels and risk of CVD outcomes in Figure 3. Results from the sensitivity analyses, including pleiotropy, heterogeneity, scatter, and funnel plots, are shown in Table 2, Supplementary Figures S1 and S2. The results from outlier detection and correction using the MR-PRESSO method are shown in Table 3.

**Figure 4:**
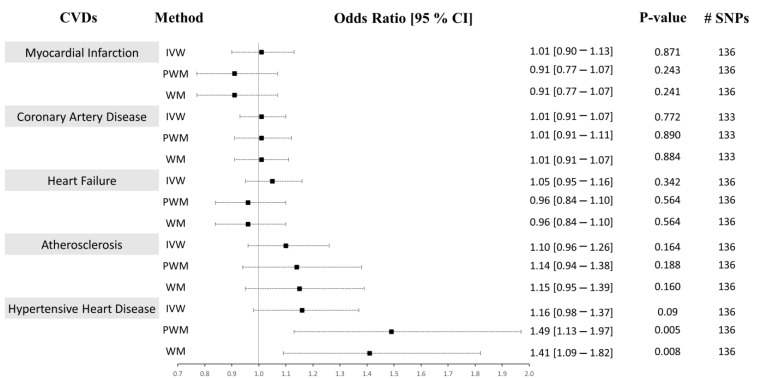
Causal associations between CRP levels and CVD using Approach 2. MR estimations were performed using IVW as the primary method and PWM and WM as sensitivity analyses. The effect sizes are shown by a black square with 95% confidence intervals as black dashed lines. The number of SNPs found in the outcome GWAS is shown in the #SNPs column. Abbreviations: cardiovascular disease: CVD, inverse-variance weighted: IVW, weighted median: WM, penalized weighted median: PWM, 95% confidence interval: 95% CI.

In Approach 2, of the 179 IVs, 42 were excluded as they were reported to have associations with either the confounders or the outcomes at a genome-wide significance level (Supplementary Table S3). The MR-PRESSO method was used to detect and correct for any outlier estimates. The 137 CRP-associated variants had F-statistics > 10 and were consolidated to be used as combined IVs in the MR analysis (F-statistics combined = 126.58). A similar strategy as above was performed to run the univariable MR analysis. We present the univariable MR results using the IVM, WM, and PWM methods between the CRP levels and risk of CVD outcomes in Figure 4. The results from the sensitivity analyses, including pleiotropy, heterogeneity, scatter, and funnel plots, are shown in Table 4, Supplementary Figures S3 and S4. The results from outlier detection and correction using the MR-PRESSO method are shown in Table 5.

### 2.1. CRP Levels Do Not Causally Associate with CAD, MI, HF, or Atherosclerosis

We analyzed the causal associations between CRP and the risk of the most prevalent CVD outcomes: myocardial infarction, coronary artery disease, heart failure, and atherosclerosis (Figure 3 and Figure 4, Supplementary Figures S1 and S3, panels A–D). In Approach 1, 178 IVs, of which 8 were LD proxies, were identified in the outcome GWAS except for CAD, where 171 instruments, of which 12 were LD proxies, were identified in the GWAS of that outcome. The heterogeneity (Cochran’s Q statistic test; Het *p*) and pleiotropy (MR-Egger Intercept that evaluates the magnitude of horizontal pleiotropy; Pleiotropy *p*) were calculated for each exposure–outcome analysis and are presented in Table 2. In Approach 2, 136 IVs, of which 8 were LD proxies, were identified in the outcome GWAS except for CAD, where 133 instruments, of which 9 were LD proxies, were identified in the GWAS of that outcome.

#### 2.1.1. Myocardial Infarction (MI)

Using both Approach 1 and 2, no associations were found between CRP and MI using the IVM and PWM method (IVW: (OR = 0.99 [0.90–1.09], *p* = 0.865), (OR = 1.01 [0.90–1.13], *p* = 0.871), PWM: (OR = 0.89 [0.80–1.00], *p* = 0.050), (OR = 0.91 [0.77–1.07], *p* = 0.243). However, using Approach 1, the WM method revealed an inverse relationship between CRP levels and risk of MI (OR = 0.89 [0.81–0.97], *p* = 0.011), but no association was seen using Approach 2 (OR = 0.91 [0.77–1.07], *p* = 0.241) (Figure 3 and Figure 4). The sensitivity analysis could explain this negative association, where Cochran’s Q test indicated significant heterogeneity (Het *p* = 4.67 × 10^−8^), and the MR-Egger method revealed a trend towards significant pleiotropy (Pleiotropy *p* = 0.055). In Approach 2, non-significant pleiotropy *p* (0.199), but significant Het *p* (3.49 × 10^−4^) were seen. The scatter and funnel plots showed evidence of asymmetry (Supplementary Figures S1–S4, panel A). The estimated causal effects for the raw and outlier-corrected MR-PRESSO analyses are shown in Table 3 and Table 5. Overall, the results revealed no relationship between CRP and MI.

#### 2.1.2. Coronary Artery Disease (CAD)

The IVW, PWM, and WM MR methods were in agreement and revealed no associations between variants affecting serum CRP levels and CAD (Approach 1: (OR = 0.99 [0.91–1.07], *p* = 0.770), (OR = 1.01 [0.91–1.11], *p* = 0.896), (OR = 0.95 [0.85–1.06], *p* = 0.335); Approach 2: (OR = 1.01 [0.91–1.07], *p* = 0.772), (OR = 1.01 [0.91–1.07], *p* = 0.890), (OR = 1.01 [0.91–1.07], *p* = 0.884)), respectively (Figure 3 and Figure 4). The Het *p* (1.54 × 10^−34^) and Pleiotropy *p* (0.005) were significant for CAD in Approach 1, indicating considerable heterogeneity and evidence of significant directional pleiotropy, which are both violations of the MR method. However, in Approach 2, Het *p* (1.01 × 10^−10^) was significant, but not pleiotropy *p* (0.614). Asymmetry was found in the funnel plot (Supplementary Figures S1–S4, panel B). The estimated causal effects for the raw and outlier-corrected MR-PRESSO analyses are shown in Table 3 and Table 5. The analyses support a lack of association between CRP and CAD.

#### 2.1.3. Heart Failure (HF)

Using the IVW MR method, we observed that higher CRP levels increase the risk of HF by 9% (OR = 1.09 [1.01–1.18], *p* = 0.023) in Approach 1, but not in Approach 2 (OR = 1.05 [0.95–1.16], *p* = 0.342). Upon performing further sensitivity analyses using the PWM and WM method, we found that the association between CRP levels and the risk of HF becomes void (Approach 1: (OR = 1.02 [0.92–1.14], *p* = 0.672), (OR = 1.05 [0.98–1.14], *p* = 0.180); Approach 2: (OR = 0.96 [0.84–1.10], *p* = 0.564), (OR = 0.96 [0.84–1.10], *p* = 0.564)) (Figure 3 and Figure 4). The Het *p* (7.81 × 10^−4^, 0.007) and Pleiotropy *p* (0.024, 0.010) were significant, indicating remarkable heterogeneity and pleiotropy. Funnel plots were also unbalanced (Supplementary Figures S1–S4, panel C). In Approach 1, the raw and outlier-corrected MR-PRESSO results were congruent and lacked association. No outliers were detected using Approach 2; thus, an outlier-corrected analysis was not performed. Our results indicate that CRP does not causally associate with HF.

#### 2.1.4. Atherosclerosis

The results for atherosclerosis were similar to the results for HF outcome. The IVW method revealed a direct association between increased circulating CRP levels and increased risk of atherosclerosis by 17% (OR = 1.17 [1.05–1.30], *p* = 0.005) in Approach 1, but not in Approach 2 (OR = 1.10 [0.96–1.26], *p* = 0.164). Nevertheless, further sensitivity analysis using the PWM and WM method eliminated this association (Approach 1: (OR = 1.15 [0.99–1.34], *p* = 0.073), (OR = 1.04 [0.92–1.17], *p* = 0.545); Approach 2: (OR = 1.14 [0.94–1.38], *p* = 0.188), (OR = 1.15 [0.95–1.39], *p* = 0.160)) (Figure 3 and Figure 4). The Het *p* was significant (5.37 × 10^−7^, 0.004), indicating considerable heterogeneity. However, the Pleiotropy *p* was insignificant (0.253, 0.068), indicating a lack of pleiotropy. The funnel plot showed no asymmetry (Supplementary Figures S1–S4, panel D). The outlier-corrected MR-PRESSO method indicates a positive relationship between CRP and atherosclerosis in Approach 1, but not in Approach 2. In conclusion, the results show limited evidence of causal association. 

### 2.2. Genetically Determined CRP Serum Levels Possibly Increase the Risk of Hypertensive Heart Disease (HHD)

The initial MR assessment provided evidence for positive causal associations between CRP and the risk of HHD (Figure 3 and Figure 4). In Approach 1, 178 instrumental variables were identified in the outcome of HHD GWAS, of which 8 were LD proxies. Using the IVW method in Approach 1, we observed that higher CRP serum levels significantly increased the risk of HHD by 21% (odds ratio (OR) = 1.21 [1.07–1.37], *p* = 0.003). Sensitivity analysis also supported the direct positive association between high circulating serum levels of CRP and an increase in the risk of HHD ((PWM method: OR = 1.37 [1.13–1.65], *p* = 0.001), (WM method: OR = 1.23 [1.06–1.44], *p* = 0.009)). Even though the IVW results from Approach 2 indicated a lack of association between CRP and HHD (OR = 1.16 [0.98–1.37], *p* = 0.09), the sensitivity results were congruent with the results from Approach 1 (OR = 1.49 [1.13–1.97], *p* = 0.005, OR = 1.41 [1.09–1.82], *p* = 0.008). In Approach 1, the raw and outlier-corrected MR-PRESSO results were congruent and indicated that higher CRP levels increase the risk of HHD (Table 3). In Approach 2, an outlier-corrected analysis was not performed as no outliers were detected (Table 5). The MR-Egger Pleiotropy *p* was insignificant (0.404, 0.123), indicating an absence of directional pleiotropy. Directional pleiotropic effects did not influence the causal association between CRP and HHD, which validates one of the main assumptions of the MR method.

**Figure 5:**
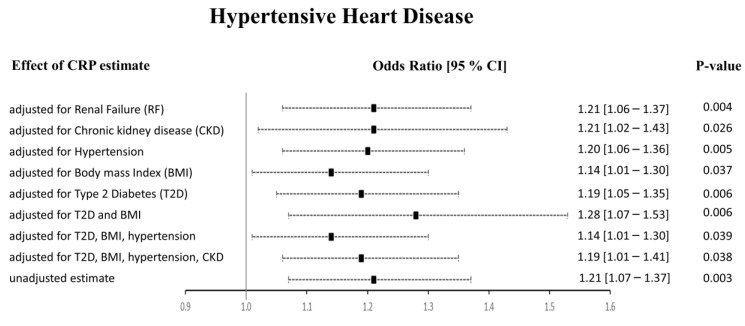
Results from the multivariable MR study between CRP and HHD. The adjusted effect sizes are shown as black squares with 95% confidence intervals presented as black dashed lines.

The Het *p* (0.007) was significant for CRP-HHD analysis in Approach 1, but not Approach 2 (0.052). We found the funnel plot to be symmetrical (Supplementary Figures S1–S4, panel E). Thus, to check whether the heterogeneity effects influenced our HHD risk estimates, we carried out multivariable Mendelian randomization (MVMR) analyses to adjust for the potential risk factors of HHD (Figure 5). We noticed that the pleiotropic SNPs were mainly associated with BMI, T2D, hypertension, and renal failure using the PhenoScanner tool. Therefore, we performed regression-based MVMR to obtain estimates independent of these risk factors. The significance and direction of causation between circulating CRP levels and HHD risk remained unchanged in the MVMR analysis when adjusted for risk factors individually or in combination (*p* < 0.05). When adjusted for only renal failure (RF) or chronic kidney disease (CKD), the odds ratio remained unaltered; however, the 95% confidence intervals increased (OR [95% CI]: 1.21 [1.06–1.37], and 1.21 [1.02–1.43], respectively). Similarly, adjusting the estimates for hypertension alone did not change the risk of the HHD outcome (1.20 [1.06–1.36]). Upon adjustment for body mass index (BMI), the OR changed to 1.14 [1.01–1.30], while the OR slightly changed from 1.21 [1.07–1.37] to 1.19 [1.05–1.35] upon adjustment for type 2 diabetes (T2D). The MVMR approach adjusted for a combination of risk factors also agreed with the preliminary analysis. It showed that higher CRP levels directly relate to HHD and increase its risk. When we adjusted the CRP-HHD outcome estimate by regressing for T2D and BMI, the OR was found to be 1.28 [1.07–1.53], *p* = 0.006; the OR became 1.14 [1.01–1.30], *p* = 0.039 when hypertension was added to this adjustment, while adding hypertension and CKD readjusted the OR to 1.19 [1.01–1.41], *p* = 0.038.

Overall, our results indicate a causality between genetically determined CRP serum levels and increased risk of HHD. Our results show that adjustment for potential risk factors such as renal failure, CKD, hypertension, BMI, and T2D separately or in combination did not alter these findings using Approach 1. In Approach 2, when the PhenoScanner tool was used to identify and exclude the SNPs associated with outcomes, we observed no association between CRP and HHD using the IVW method. However, the WM and PWM results remained congruent with the results from Approach 1, showing a positive relationship between CRP and HHD risk. 

### 2.3. CVD Does Not Causally Associate with CRP

We also studied the effect of reverse causation between CVD and CRP using the IVW two-sample MR method (Table 6). A similar methodology, as previously explained, was followed. We found varying numbers of genetic determinants in each of the exposure–outcome datasets. Seven SNPs were found in the MI-CRP dataset, of which one was an LD proxy. Thirty-eight SNPs were found for the CAD-CRP dataset, of which one was an LD proxy. For HF and HHD, two SNPs, none of which were proxies, were found in their respective outcomes. Only one SNP was found in the atherosclerosis-CRP outcome GWAS; thus, the Wald ratio MR method was used to determine the causal estimates. The heterogeneity, but not pleiotropy, was significant for the MI-CRP outcome. Insignificant pleiotropy was found in the CAD-CRP outcome. For HF, atherosclerosis, and HHD, the pleiotropy or heterogeneity values could not be determined, as the analyses were underpowered. Based on the OR (95% CI values), CVD is not causally associated with CRP except for atherosclerosis. However, the causal estimate for atherosclerosis-CRP was based only on a single SNP analysis (rs1980496). Using the PhenoScanner tool, we found that rs1980496 associates with T2D at a genome-wide significance level. Since patients with T2D have been shown to have higher CRP levels [32], this SNP should be excluded from the atherosclerosis-CRP analysis, making the relationship between atherosclerosis and CRP unreliable. In conclusion, we found no evidence that CVD causally leads to changes in CRP levels.

## 3. Discussion

In this study, we evaluated the effects of genetic variants associated with serum C-reactive protein (CRP) and the risk of five cardiovascular diseases (CVD) using comprehensive two-sample bidirectional Mendelian randomization (MR) analysis. We show, for the first time, a direct causality between higher levels of CRP and a higher risk of hypertensive heart disease (HHD). We also found that circulating CRP levels are causally not associated with the risk of myocardial infarction (MI), coronary artery disease (CAD), heart failure (HF), or atherosclerosis. Moreover, our results corroborate findings from other studies showing that higher CRP levels do not have a causal relationship with the adjusted risk of CAD [24,26,28], HF [27], atherosclerosis [26,30], or MI [29]. Our highly powered analysis and other MR studies show that CRP is unlikely to play a credible causal role in the risk of certain types of heart diseases. We also demonstrated that CVD is unlikely to play a role in increasing CRP levels. However, more powered analyses are required to confirm the lack of reverse causation.

Markers of inflammation, such as CRP, are prominent predictors of elevated blood pressure, and a few observational studies have confirmed their role in the development of hypertension [23]. Multiple human and animal studies confirm the role of inflammation, as predicted by plasma CRP level, in the development of hypertension [22]. Another publication showed a marginal significance supporting the causality between CRP and diastolic blood pressure [23]. A separate study revealed correlations between CRP and pulse pressure, systolic blood pressure, and hypertension. However, when confounding factors were taken into account, the causal relationship was no longer present [33]. The process of vascular remodeling, which results in hypertension, consists of complex interactions between multiple systems, including the immune system, the renin–angiotensin system, the vascular system, and the central nervous system [34,35]. Plasma CRP levels reflect systemic inflammation and could represent an important marker for the development and progress of hypertension [18]. HHD develops over time in people with high blood pressure (hypertension) [36]. Since CRP is known to be associated with hypertension, which, in the long run, leads to HHD, we explored the association between CRP and HHD.

Using different approaches in the MR study, we found evidence that higher CRP levels increase the risk of HHD. While inflammation is closely intertwined with the development of hypertension and HHD, a probable relationship exists between CRP and HHD, as shown in our extensive sensitivity and multivariable analyses. The initial Mendelian randomization (MR), sensitivity, and multivariable MR (MVMR) analyses confirmed a positive association between high CRP levels and increased HHD risk in Approach 1. One of the concerns in the MR approach is the possibility of the genetic variants being associated with a confounder [37]. Confounders can affect the risk of CVD via a pathway independent of exposure (CRP), which is one of the violations of the MR assumptions. For example, higher BMI affects blood pressure and increases the risk of CVDs [38]. RF and CKD patients often need dialysis because of incompetent kidneys, and these conditions are associated with accelerated vasculopathy and premature hypertension [39]. Individuals suffering from T2D are also at a higher risk of CVD events due to accelerated atherosclerosis [40,41]. Since we found heterogeneity in our MR sensitivity results, we also evaluated the effects of these confounders on the risk of the outcome. Based on the results from the sensitivity analyses, we showed that CRP has an independent causal association with the increased risk of HHD. This causal relationship is not dependent on any confounding effects from hypertension, body mass index, chronic kidney disease (CKD), renal failure (RF), and type 2 diabetes (T2D) individually or in combination. Another tool used to identify IVs associated with confounders is PhenoScanner. In Approach 2, we found the WM and PWM methods to agree with the initial analysis from Approach 1. However, we found that the IVW method differs in its risk prediction. Updated MR studies are required to confirm the role of CRP in the prediction of the risk of HHD.

To our knowledge, this is the first report of a novel causal relationship between an inflammatory factor, CRP, and the risk of HHD. The results obtained from our study could be attributed to the large sample size and an optimal study design incorporating robust MR and sensitivity methods. We used two different approaches to check for potential outliers, which makes the results more reliable. The MR-PRESSO test is a widely accepted method to identify and correct for probable biases arising from horizontal pleiotropy [42]. In addition to the MR-PRESSO method, we also used the PhenoScanner tool to eliminate IVs associated with confounders. Further, including variants not just in the *CRP* gene locus, but also in those genes that modulate the circulating levels of CRP added to our advantage. We chose SNPs associated with CRP at a genome-wide level, thus collaborating reliable association with the exposure. We also obtained independent SNPs using LD to obtain valid inference in the MR methods. Further pruning of variants having intermediate allele frequencies allowed us to determine the reliability of the IVs. The individual and the F-statistics combined value of all IVs was >10, indicating a strong association with the exposure. Based on these strict inclusion criteria, our study was not subjected to weak instrument bias [31]. However, our study has the following limitations: firstly, we performed an analysis that only included participants of European ancestry; thus, it is still to be determined whether these associations can be extrapolated or generalized to individuals belonging to non-European ancestries. Moreover, we did not have access to individual-level data and used summary statistics in our MR analyses, which may have introduced unobserved bias. Based on the differences in the IVW results of Approaches 1 and 2, our findings warrant updated MR studies to confirm the role of CRP as a clinical biomarker for HHD. Lastly, we did not evaluate how CRP levels affected the potential confounders or study the effect of any indirect pathways altering the risk of HHD. Despite these limitations, our comprehensive and robust methods provide strong evidence for the probable relationship between CRP levels and HHD. This finding can be leveraged in future interventional and therapeutic studies.

## 4. Materials and Methods

### 4.1. Study Design

We performed a comprehensive two-sample bidirectional Mendelian randomization (MR) study [43] to investigate the causality between C-reactive protein (CRP) levels and cardiovascular disease (CVD) (Figure 1A and Supplementary Table S1). We evaluated CVDs such as hypertensive heart disease (HHD), heart failure (HF), and myocardial infarction (MI). Even though atherosclerosis is a substrate of coronary artery disease (CAD), it is still the leading cause of vascular disease, where inflammation plays a significant role. Therefore, we wanted to evaluate the risk of both atherosclerosis and CAD. We used two independent datasets that included participants from different geographical regions to study the risk of atherosclerosis or CAD. The validity of the MR method depends on the instrumental variables (IVs) being satisfied for three key assumptions (Figure 1B): Assumption I –IVs are not associated with any confounders, Assumption II—the IVs are only robustly associated with the exposure, and Assumption III—IVs only affect the risk of the outcome through the exposure and not through any other biological pathway. Therefore, we carefully selected the candidate IVs and used multiple MR approaches to make the conclusions robust. An F-statistic < 10 is a weak IV-exposure association [44,45]; therefore, in our study, we excluded any IVs subjected to weak instrument bias (F-statistics < threshold) [46]. We also used the intercept and *p*-value from the MR-Egger regression analysis to determine the directional horizontal pleiotropy [47]. An MR-Egger intercept with a significant *p*-value (*p* < 0.05) was considered a violation of the MR study. Heterogeneity was evaluated using Cochran’s Q test. In Approach 1, the MR pleiotropy residual sum and outlier (MR-PRESSO) [42] method was used to remove any horizontal pleiotropic outlier IVs, followed by the Multivariable MR (MVMR) analysis to adjust for potential confounders. In Approach 2, the PhenoScanner tool [48] was used to exclude any IVs associated with CVD confounders at a genome-wide significance level (*p* < 5 × 10^−8^). All analyses were based on participants of European ancestry only.

### 4.2. Genetic Variants and Data Sources

#### 4.2.1. Outcome Dataset

Publicly available summary statistics from FinnGen or EBI GWAS from the MR Base GWAS Catalog were used to obtain genetic variants associated with CVD as the outcome (Supplementary Table S1). If multiple catalogs were available for extracting summary statistics for a given outcome, we chose the most recent and the one containing the most significant number of cases/controls to keep our analysis robust. For studying the risk of CAD, summary statistics from the European Bioinformatics Institute GWAS (EBI GWAS) from the MR Base catalog were obtained. Please refer to the original paper for details on the baseline characteristics of the participants and cohorts included in the CAD dataset [49]. Since UKBB was not a cohort included in the CAD GWAS, we considered the CAD dataset independent from the UKBB CRP exposure dataset for our two-sample MR study. The FinnGen and UKBB databases are recognized as being mutually exclusive. We also carefully examined the outcome’s phenotype definitions to select appropriate datasets to avoid any type 1 error rate inflation arising from sample overlap.

#### 4.2.2. Exposure Dataset

Genome-wide significant (*p* < 5 × 10^−8^) SNPs associated with serum CRP levels were obtained from the UK Biobank (UKBB) summary statistics using the MR Base GWAS Catalog (http://app.mrbase.org/ accessed on 9 January 2023) [50,51]; Supplementary Table S1). UKBB is the most extensive study to date and includes data from 343,524 participants for CRP [52]. Candidate IVs were carefully selected using quality control steps (Figure 2). Since two-sample MR methods require that the instruments be independent and do not have Linkage Disequilibrium (LD) between them, we used the ‘clump_data’ function in R available via the ‘TwoSampleMR’ package. We pruned SNPs in LD (r^2^ > 0.001) within a clumping distance of 10,000 kb. Next, we removed palindromic SNPs with intermediate allele frequencies to remove potential biases. To test the strength and validity of the IVs, we calculated F-statistics [46] for each variant and only included the variants associated with CRP at an F-statistic > 10. All effect alleles were aligned to the CRP-increasing allele.

### 4.3. Candidate IV Selection, Mendelian Randomization, and Statistical Analysis

We used two different approaches to investigate the relationship between CRP and CVD. In Approach 1, we applied the MR-PRESSO test to exclude horizontal pleiotropic outliers from the selected IVs. We conducted this analysis using the MR-PRESSO package available in R with a significance threshold cutoff of 0.05 and the number of distributions set to 10,000. If a particular exposure SNP was absent in an outcome dataset, we replaced it with a proxy SNP in LD (r^2^ > 0.8). For some of the exposure SNPs, one or more SNPs or their proxies were not found in the outcome dataset and were eliminated from the analysis. The PhenoScanner tool identified body mass index (BMI), hypertension, renal failure (RF), kidney disease, and type 2 diabetes (T2D) as potential confounders. Therefore, after performing the initial MR analysis, we used MVMR [37] to adjust for the effects of multiple risk factors on a single CVD outcome, as identified by the PhenoScanner tool. On the one hand, the traditional MR method is used to evaluate the estimate of the total exposure on the outcome; on the other hand, MVMR determines the estimate of each exposure directly on the outcome [53]. We used the ‘mv_multiple’ function in MVMR analysis using R to estimate the probable pleiotropic effects of the concomitant risk factors. In the MVMR method, all exposures were fit simultaneously against the remainder of the outcome adjusted for all of the other outcomes, i.e., a single regression model was used to adjust effects from related risk factors [53]. We obtained GWAS summary statistics from the MR Base GWAS Catalog from UKBB for BMI, hypertension, RF, and T2D; and from EBI GWAS Catalog for chronic kidney disease (CKD) and type 2 diabetes adjusted for body mass index (T2D and BMI) (Supplementary Table S2) to perform the MVMR analysis. MVMR estimates were adjusted for the individual and a combination of risk factors (Supplementary Table S2).

In Approach 2, the PhenoScanner tool (www.phenoscanner.medschl.cam.ac.uk, accessed on 9 January 2023 [48]) was used to cross-reference the IVs and exclude any SNPs associated at a genome-wide significance (*p* < 5 × 10^−8^) with potential confounders or outcomes (Supplementary Table S3). IVs absent in the outcome GWAS that cannot be replaced by a proxy SNP were excluded from the MR analysis. Next, we performed outlier IV detection and correction using the MR-PRESSO method mentioned earlier.

To study the causal relationship between CRP and CVD, we applied the MR method in R version 4.1.2 using the ‘TwoSampleMR’ package version 0.5.5. To evaluate the robustness of the estimates, we used various two-sample MR methods. For the univariable analysis, we used four MR methods: inverse-variance weighted (IVW; primary method), weighted median (WM), penalized weighted median (PWM), and MR-Egger. Multiple MR methods allow robust estimates even if potential violations are encountered in the MR approach. In addition, using different methods allows for optimal MR analysis as they differ in efficiency, limitations, and strengths [54]. The Egger method is used to detect violations of assumptions or the presence of outliers in the MR method, but it lacks precision [55,56]. On the other hand, IVW is the most efficient MR method and has the most considerable statistical power, but it assumes that all variants are valid or have no pleiotropy [55]. The WM method is robust in the presence of outliers and can provide firm estimates even when 50% of the IVs are invalid [56]. However, it depends on which genetic variants are excluded or included [55]. The PWM method carefully evaluates IV estimates based on their heterogeneous ratio estimates. The estimates from valid IVs are unaffected; however, the invalid IV estimates are gravely down-weighted by penalization [56]. Sensitivity tests for each analysis were conducted using the MR-Egger, WM, and PWM methods. We also determined pleiotropy based on the intercepts from the MR-Egger regression analysis to evaluate any partisan biases in the MR approach. Cochran’s Q statistic test using the MR-Egger method was used to determine the heterogeneity between the genetic variants [31]. We inspected the funnel and scatter plots generated using the MR tests to evaluate the outlier bias. The presence of outliers in the IVs can lead to false positive associations; thus, we used the MR-PRESSO method to outlier-correct the causal estimates in the IVW analysis. The MR-PRESSO method includes three aspects: the global test detects horizontal pleiotropy, the outlier test corrects horizontal pleiotropy by removing outliers, and the distortion test is used to significantly evaluate the variation in causal estimates before and after outlier correction. The MR-PRESSO method requires at least half of the variants to be valid IVs with a balanced pleiotropy. It also requires that the IV exposure and pleiotropic effects of the IVs be uncorrelated and satisfy the Instrument Strength Independent of Direct Effect (InSIDE) prerequisite [42].

Avoiding bias from reverse causation is also critical for a valid MR study [57]. Thus, to explore whether reverse causation could introduce any distortion, we performed a univariable MR analysis (IVW method) using different CVDs as the exposure and CRP as the outcome (Supplementary Table S1).

Estimates of CRP–CVD associations are presented as forest plots depicting the odds ratio and 95% confidence intervals of each CVD outcome per standard deviation increase in genetically predicted levels of CRP (mg/L). For all analyses, a *p* < 0.05 was considered statistically significant.

## 5. Conclusions

In summary, we demonstrate for the first time that a genetically determined increase in CRP levels increases the risk of hypertensive heart disease by 21% (OR = 1.21 [1.07–1.37]). We used two approaches to account for horizontal pleiotropies and to perform the outlier correction of beta estimates. These findings remained unaltered upon adjustment for related confounders in the MVMR analyses. We also corroborated results from other studies where serum CRP levels do not associate with adjusted risk of other CVD: myocardial infarction, coronary artery disease, heart failure, and atherosclerosis. We also found no evidence of reverse causation, i.e., CVD does not affect the levels of CRP. Our findings enhance the understanding of HHD disease etiology and prompt the need for future studies to investigate and confirm the role of CRP as a clinical biomarker that regulates the risk of HHD and its potential role in therapeutic interventions.

## Figures and Tables

**Table 1:** Characteristics of the 179 candidate SNPs predictive of C-reactive protein levels.

SNP	EA	OA	EAF	Beta	SE	*p*	CHR	POS	Sample Size	Fstats
rs429358	T	C	0.844	0.266	0.003	1.00 × 10^−200^	19	45,411,941	343,524	6647.33
rs7551731	T	C	0.670	0.172	0.003	1.00 × 10^−200^	1	159,694,779	343,524	4673.13
rs7970695	A	G	0.624	0.146	0.002	1.00 × 10^−200^	12	121,423,376	343,524	3544.54
rs2154384	C	T	0.631	0.125	0.002	1.00 × 10^−200^	1	66,108,517	343,524	2550.92
rs12133641	A	G	0.590	0.094	0.002	1.00 × 10^−200^	1	154,428,283	343,524	1507.50
rs1260326	T	C	0.393	0.074	0.002	1.00 × 10^−200^	2	27,730,940	343,524	916.40
rs17616063	A	G	0.924	0.126	0.005	2.33 × 10^−171^	16	51,436,882	343,524	779.55
rs7012637	A	G	0.475	0.048	0.002	1.29 × 10^−86^	8	9,173,209	343,524	389.34
rs4656849	G	A	0.601	0.046	0.002	8.39 × 10^−79^	1	159,723,521	343,524	353.40
rs6734238	G	A	0.402	0.043	0.002	7.74 × 10^−71^	2	113,841,030	343,524	316.80
rs1889316	C	T	0.846	0.056	0.003	2.60 × 10^−63^	1	154,352,017	343,524	282.22
rs56015600	G	A	0.630	0.040	0.002	1.48 × 10^−58^	1	247,601,886	343,524	260.40
rs1800961	C	T	0.969	0.105	0.007	2.86 × 10^−53^	20	43,042,364	343,524	236.15
rs728538	G	T	0.167	0.045	0.003	1.65 × 10^−43^	16	51,205,819	343,524	191.35
rs2239222	G	A	0.349	0.034	0.003	4.78 × 10^−42^	14	73,011,885	343,524	184.66
rs6519133	T	C	0.607	0.033	0.002	5.73 × 10^−42^	22	39,096,602	343,524	184.30
rs1490384	C	T	0.502	0.031	0.002	2.73 × 10^−38^	6	126,851,160	343,524	167.45
rs35371668	T	C	0.181	0.041	0.003	6.19 × 10^−38^	6	32,561,638	343,524	165.82
rs149624078	C	T	0.986	0.130	0.010	1.69 × 10^−35^	15	53,728,710	343,524	154.69
rs11265157	G	C	0.260	0.033	0.003	1.05 × 10^−33^	1	159,224,029	343,524	146.45
rs28929474	C	T	0.980	0.100	0.008	2.19 × 10^−32^	14	94,844,947	343,524	140.42
rs2836881	G	T	0.733	0.032	0.003	8.98 × 10^−32^	21	40,466,299	343,524	137.62
rs469802	A	G	0.796	0.035	0.003	1.04 × 10^−31^	1	91,545,826	343,524	137.33
rs2393794	C	T	0.186	0.036	0.003	3.90 × 10^−31^	12	121,378,976	343,524	134.69
rs75460349	A	C	0.976	0.091	0.008	3.77 × 10^−30^	1	27,180,088	343,524	130.19
rs6486122	T	C	0.691	0.029	0.003	1.34 × 10^−28^	11	13,361,524	343,524	123.09
rs12944581	C	G	0.730	0.031	0.003	2.89 × 10^−28^	17	76,348,830	343,524	121.58
rs12231235	G	A	0.573	0.027	0.002	4.96 × 10^−28^	12	95,857,690	343,524	120.50
rs115785198	T	C	0.071	0.051	0.005	1.60 × 10^−27^	19	45,249,509	343,524	118.18
rs3768321	T	G	0.197	0.032	0.003	4.96 × 10^−27^	1	40,035,928	343,524	115.93
rs7828742	G	A	0.598	0.026	0.002	5.94 × 10^−27^	8	116,960,729	343,524	115.58
rs635634	T	C	0.184	0.033	0.003	3.19 × 10^−26^	9	136,155,000	343,524	112.24
rs1037170	T	C	0.722	0.028	0.003	1.64 × 10^−25^	17	72,702,914	343,524	109.00
rs2246833	T	C	0.340	0.026	0.003	1.65 × 10^−25^	10	91,005,854	343,524	108.99
rs55855238	C	T	0.651	0.026	0.003	1.91 × 10^−25^	18	55,089,715	343,524	108.70
rs204914	T	C	0.047	0.059	0.006	3.07 × 10^−25^	19	45,466,335	343,524	107.75
rs150844304	C	A	0.025	0.080	0.008	3.38 × 10^−25^	15	43,726,625	343,524	107.56
rs10769254	G	C	0.810	0.031	0.003	1.79 × 10^−24^	11	47,362,465	343,524	104.26
rs35511257	C	G	0.095	0.043	0.004	2.82 × 10^−23^	6	32,545,392	343,524	98.80
rs13242809	T	A	0.726	0.027	0.003	4.31 × 10^−23^	7	22,746,564	343,524	97.96
rs62217799	T	G	0.657	0.025	0.003	3.98 × 10^−22^	20	62,347,191	343,524	93.55
rs17138478	A	C	0.129	0.034	0.004	5.62 × 10^−22^	17	36,073,320	343,524	92.87
rs185320691	C	G	0.107	0.041	0.004	5.96 × 10^−22^	6	32,490,292	343,524	92.75
rs4655802	G	A	0.410	0.023	0.002	6.11 × 10^−22^	1	65,888,231	343,524	92.70
rs2395470	A	G	0.329	0.024	0.003	1.15 × 10^−21^	6	31,235,261	343,524	91.44
rs61542988	C	T	0.754	0.026	0.003	1.99 × 10^−21^	7	22,882,291	343,524	90.36
rs1441171	G	T	0.476	0.023	0.002	5.17 × 10^−21^	2	214,033,637	343,524	88.47
rs11777625	C	T	0.546	0.022	0.002	8.48 × 10^−21^	8	126,333,642	343,524	87.50
rs141783576	G	C	0.927	0.043	0.005	1.82 × 10^−20^	6	127,439,897	343,524	85.99
rs687339	C	T	0.228	0.026	0.003	3.07 × 10^−20^	3	135,932,359	343,524	84.95
rs2847289	C	A	0.572	0.022	0.002	3.36 × 10^−20^	18	12,812,167	343,524	84.78
rs73001065	C	G	0.071	0.043	0.005	3.99 × 10^−20^	19	19,460,541	343,524	84.43
rs112875651	G	A	0.607	0.022	0.002	1.32 × 10^−19^	8	126,506,694	343,524	82.08
rs12496226	T	G	0.321	0.023	0.003	1.37 × 10^−19^	3	49,734,040	343,524	82.01
rs2161037	A	G	0.545	0.022	0.002	2.32 × 10^−19^	2	169,893,419	343,524	80.96
rs2700938	C	T	0.376	0.022	0.002	3.78 × 10^−19^	7	36,085,142	343,524	79.99
rs7846549	G	A	0.896	0.035	0.004	4.09 × 10^−19^	8	9,239,371	343,524	79.83
rs714052	A	G	0.875	0.031	0.004	3.38 × 10^−18^	7	72,864,869	343,524	75.66
rs7357754	G	A	0.500	0.021	0.002	4.14 × 10^−18^	9	92,207,308	343,524	75.26
rs12300845	G	T	0.967	0.058	0.007	4.55 × 10^−18^	12	24,195,798	343,524	75.07
rs77704739	T	C	0.958	0.051	0.006	6.65 × 10^−18^	5	52,080,909	343,524	74.33
rs8178824	T	C	0.030	0.060	0.007	1.31 × 10^−17^	17	64,224,775	343,524	72.99
rs9638882	C	A	0.791	0.024	0.003	1.24 × 10^−16^	7	1,023,617	343,524	68.56
rs11118625	A	G	0.731	0.022	0.003	4.12 × 10^−16^	1	221,103,388	343,524	66.18
rs28429148	A	G	0.434	0.020	0.002	4.60 × 10^−16^	16	53,798,319	343,524	65.97
rs6961634	G	A	0.851	0.027	0.003	6.82 × 10^−16^	7	99,181,839	343,524	65.19
rs1800693	T	C	0.599	0.020	0.002	7.10 × 10^−16^	12	6,440,009	343,524	65.11
rs11605427	G	C	0.598	0.020	0.002	8.26 × 10^−16^	11	59,928,672	343,524	64.81
rs340023	T	C	0.269	0.022	0.003	8.45 × 10^−16^	15	60,908,082	343,524	64.77
rs9604045	G	T	0.750	0.023	0.003	1.36 × 10^−15^	13	113,927,208	343,524	63.84
rs1933736	C	T	0.401	0.019	0.002	6.28 × 10^−15^	6	116,387,255	343,524	60.81
rs704017	A	G	0.428	0.019	0.002	1.36 × 10^−14^	10	80,819,132	343,524	59.29
rs34139656	G	A	0.328	0.020	0.003	1.39 × 10^−14^	16	88,534,923	343,524	59.26
rs8060025	T	G	0.389	0.019	0.002	3.87 × 10^−14^	16	27,327,214	343,524	57.24
rs11145763	C	A	0.433	0.018	0.002	4.16 × 10^−14^	9	139,263,596	343,524	57.09
rs9738365	A	C	0.267	0.020	0.003	5.94 × 10^−14^	12	31,997,635	343,524	56.40
rs2049045	G	C	0.813	0.023	0.003	7.03 × 10^−14^	11	27,694,241	343,524	56.06
rs7084062	G	A	0.489	0.018	0.002	8.85 × 10^−14^	10	133,736,636	343,524	55.61
rs6920220	A	G	0.223	0.021	0.003	9.11 × 10^−14^	6	138,006,504	343,524	55.56
rs4764939	C	T	0.531	0.018	0.002	9.33 × 10^−16^	12	103,522,952	343,524	55.51
rs519790	G	C	0.342	0.019	0.003	1.34 × 10^−13^	11	72,504,141	343,524	54.79
rs6905544	G	A	0.600	0.018	0.002	1.51 × 10^−13^	6	98,411,631	343,524	54.56
rs34298354	C	T	0.878	0.027	0.004	1.97 × 10^−13^	1	247,588,053	343,524	54.04
rs3935032	C	T	0.623	0.018	0.003	1.98 × 10^−13^	1	1,564,194	343,524	54.03
rs1905505	A	G	0.284	0.019	0.003	2.06 × 10^−13^	3	170,695,426	343,524	53.95
rs2250010	T	C	0.810	0.022	0.003	2.72 × 10^−13^	12	47,193,818	343,524	53.41
rs73656635	A	C	0.154	0.024	0.003	8.63 × 10^−13^	9	104,117,630	343,524	51.14
rs799260	G	A	0.834	0.023	0.003	9.08 × 10^−13^	12	56,921,304	343,524	51.04
rs185575165	A	T	0.280	0.019	0.003	1.06 × 10^−12^	18	57,863,231	343,524	50.73
rs4704093	G	T	0.468	0.017	0.002	1.27 × 10^−12^	5	72,976,175	343,524	50.38
rs1476698	G	A	0.369	0.018	0.002	1.28 × 10^−12^	2	242,296,449	343,524	50.37
rs1800919	A	C	0.237	0.020	0.003	2.27 × 10^−12^	2	102,759,293	343,524	49.24
rs17652767	G	A	0.894	0.027	0.004	3.48 × 10^−12^	15	53,169,284	343,524	48.40
rs2161374	C	T	0.513	0.017	0.002	4.28 × 10^−12^	5	172,176,886	343,524	47.99
rs112303588	C	G	0.033	0.049	0.007	4.51 × 10^−12^	12	121,706,242	343,524	47.89
rs17781691	A	G	0.506	0.017	0.002	4.56 × 10^−12^	14	73,366,493	343,524	47.87
rs7311631	A	G	0.507	0.016	0.002	5.05 × 10^−12^	12	90,429,028	343,524	47.67
rs12540285	A	G	0.780	0.020	0.003	6.43 × 10^−12^	7	15,031,2474	343,524	47.19
rs1545536	C	T	0.780	0.020	0.003	8.01 × 10^−12^	8	144,643,169	343,524	46.77
rs4018180	G	A	0.932	0.033	0.005	8.05 × 10^−12^	16	2,169,458	343,524	46.75
rs10760691	G	A	0.383	0.017	0.003	8.90 × 10^−12^	9	102,281,383	343,524	46.56
rs4790286	T	A	0.781	0.020	0.003	1.30 × 10^−11^	17	1,652,483	343,524	45.81
rs1985157	C	T	0.413	0.016	0.002	1.68 × 10^−11^	19	18,513,594	343,524	45.31
rs6792725	A	G	0.307	0.018	0.003	1.96 × 10^−11^	3	24,520,283	343,524	45.02
rs1348675	A	G	0.233	0.019	0.003	2.12 × 10^−11^	13	58,712,638	343,524	44.86
rs4609871	T	C	0.552	0.016	0.002	2.66 × 10^−11^	16	29,932,064	343,524	44.41
rs12132412	G	A	0.388	0.016	0.002	3.38 × 10^−11^	1	21,820,042	343,524	43.94
rs79225028	C	G	0.895	0.026	0.004	3.58 × 10^−11^	12	120,869,983	343,524	43.83
rs2050392	G	A	0.388	0.016	0.002	4.16 × 10^−11^	10	30,691,503	343,524	43.54
rs7652415	T	C	0.128	0.023	0.004	5.19 × 10^−11^	3	9,505,238	343,524	43.11
rs1883711	C	G	0.031	0.046	0.007	5.31 × 10^−11^	20	39,179,822	343,524	43.06
rs2110944	C	T	0.531	0.016	0.002	5.91 × 10^−11^	2	37,090,233	343,524	42.85
rs72694393	G	T	0.519	0.016	0.002	6.38 × 10^−11^	14	24,874,193	343,524	42.71
rs6083801	C	T	0.473	0.016	0.002	6.53 × 10^−11^	20	25,307,574	343,524	42.66
rs57014345	G	T	0.602	0.016	0.002	6.60 × 10^−11^	6	7,010,430	343,524	42.64
rs11850396	G	A	0.640	0.016	0.002	7.13 × 10^−11^	14	96,919,620	343,524	42.49
rs7956514	G	T	0.287	0.017	0.003	1.16 × 10^−10^	12	12,879,254	343,524	41.53
rs12941913	T	C	0.608	0.016	0.002	2.12 × 10^−10^	17	1,346,417	343,524	40.35
rs762360	C	T	0.301	0.017	0.003	2.49 × 10^−10^	21	37,425,955	343,524	40.04
rs60987662	G	A	0.380	0.015	0.002	3.52 × 10^−10^	7	101,844,851	343,524	39.37
rs653170	T	C	0.359	0.016	0.002	3.96 × 10^−10^	1	112,328,245	343,524	39.14
rs142296998	A	G	0.006	0.100	0.016	4.28 × 10^−10^	20	60,888,267	343,524	38.98
rs10175899	A	T	0.673	0.016	0.003	5.76 × 10^−10^	2	113,801,150	343,524	38.40
rs6012927	G	A	0.358	0.016	0.003	6.05 × 10^−10^	20	49,036,638	343,524	38.31
rs11577023	T	C	0.690	0.016	0.003	6.12 × 10^−10^	1	222,061,973	343,524	38.29
rs7563362	G	A	0.858	0.021	0.003	8.01 × 10^−10^	2	620,297	343,524	37.76
rs10831676	A	C	0.539	0.015	0.002	8.39 × 10^−10^	11	11,820,449	343,524	37.67
rs13013	A	C	0.584	0.015	0.002	9.15 × 10^−10^	10	75,562,161	343,524	37.50
rs73137144	A	G	0.807	0.019	0.003	9.80 × 10^−10^	7	74,073,590	343,524	37.37
rs6686095	T	G	0.811	0.019	0.003	1.02 × 10^−9^	1	93,883,993	343,524	37.29
rs3746778	G	A	0.580	0.015	0.002	1.03 × 10^−9^	20	61,341,472	343,524	37.27
rs117211511	A	G	0.044	0.036	0.006	1.07 × 10^−9^	12	121,701,433	343,524	37.19
rs1223801	G	A	0.163	0.020	0.003	1.11 × 10^−9^	1	214,348,141	343,524	37.13
rs13062093	G	T	0.366	0.015	0.002	1.44 × 10^−9^	3	35,667,057	343,524	36.62
rs1800973	A	C	0.061	0.030	0.005	1.85 × 10^−9^	12	69,744,014	343,524	36.13
rs28472312	T	C	0.704	0.016	0.003	1.98 × 10^−9^	16	28,826,049	343,524	36.00
rs12992995	C	A	0.724	0.016	0.003	3.44 × 10^−9^	2	175,197,545	343,524	34.92
rs62104180	G	A	0.950	0.032	0.005	3.87 × 10^−9^	2	466,003	343,524	34.69
rs114414824	T	C	0.959	0.036	0.006	4.01 × 10^−9^	2	113,995,296	343,524	34.62
rs12516176	C	T	0.274	0.016	0.003	4.17 × 10^−9^	5	150,435,645	343,524	34.55
rs11693537	A	G	0.419	0.014	0.002	4.21 × 10^−9^	2	102,541,080	343,524	34.53
rs11708067	G	A	0.246	0.016	0.003	4.41 × 10^−9^	3	123,065,778	343,524	34.44
rs76040012	T	C	0.898	0.023	0.004	4.44 × 10^−9^	2	59,150,981	343,524	34.42
rs2030291	A	T	0.612	0.014	0.002	4.66 × 10^−9^	11	16,251,251	343,524	34.33
rs2166625	G	C	0.628	0.014	0.002	5.84 × 10^−9^	13	42,584,871	343,524	33.89
rs75064168	A	G	0.091	0.024	0.004	6.22 × 10^−9^	3	101,845,939	343,524	33.76
rs4006577	G	A	0.379	0.014	0.002	6.54 × 10^−9^	1	236,301,301	343,524	33.67
rs72636674	C	G	0.166	0.019	0.003	7.04 × 10^−9^	4	67,998,903	343,524	33.53
rs17308476	T	C	0.111	0.022	0.004	7.11 × 10^−9^	2	174,868,755	343,524	33.51
rs7833554	T	C	0.555	0.014	0.002	7.26 × 10^−9^	8	103,642,986	343,524	33.47
rs7250946	T	C	0.044	0.034	0.006	7.65 × 10^−9^	19	45,140,020	343,524	33.36
rs1580300	T	A	0.197	0.017	0.003	8.02 × 10^−9^	12	21,701,150	343,524	33.27
rs9838974	A	G	0.410	0.014	0.002	9.49 × 10^−9^	3	136,987,413	343,524	32.94
rs2432195	T	C	0.827	0.018	0.003	9.68 × 10^−9^	5	56,120,413	343,524	32.91
rs12620844	C	T	0.431	0.014	0.002	1.08 × 10^−8^	2	232,324,510	343,524	32.70
rs654912	T	C	0.308	0.015	0.003	1.10 × 10^−8^	6	138,185,422	343,524	32.66
rs4148155	A	G	0.886	0.021	0.004	1.42 × 10^−8^	4	89,054,667	343,524	32.16
rs3125326	C	A	0.609	0.014	0.002	1.45 × 10^−8^	10	63,053,788	343,524	32.13
rs7317323	C	T	0.057	0.029	0.005	1.52 × 10^−8^	13	42,957,489	343,524	32.03
rs12992747	A	C	0.228	0.016	0.003	1.66 × 10^−8^	2	178,190,491	343,524	31.86
rs889745	T	G	0.524	0.014	0.002	1.67 × 10^−8^	16	79,042,774	343,524	31.85
rs1859726	C	T	0.093	0.023	0.004	1.66 × 10^−8^	1	44,396,215	343,524	31.85
rs9964912	A	G	0.571	0.014	0.002	1.79 × 10^−8^	18	13,002,309	343,524	31.71
rs150649461	C	G	0.015	0.056	0.010	1.94 × 10^−8^	1	92,925,654	343,524	31.55
rs34761529	C	T	0.795	0.017	0.003	2.20 × 10^−8^	1	22,681,214	343,524	31.31
rs11617494	A	G	0.241	0.016	0.003	2.23 × 10^−8^	13	110,383,798	343,524	31.28
rs2608101	C	T	0.701	0.015	0.003	2.35 × 10^−8^	5	170,516,570	343,524	31.19
rs80292319	T	C	0.941	0.028	0.005	3.03 × 10^−8^	15	76,508,632	343,524	30.69
rs141179989	C	T	0.983	0.055	0.010	3.13 × 10^−8^	16	51,067,084	343,524	30.63
rs6845703	A	G	0.662	0.014	0.003	3.50 × 10^−8^	4	45,134,713	343,524	30.41
rs35816944	A	G	0.007	0.081	0.015	3.54 × 10^−8^	16	1,828,030	343,524	30.39
rs2172131	T	C	0.420	0.013	0.002	3.66 × 10^−8^	10	133,978,962	343,524	30.33
rs7537072	T	C	0.584	0.013	0.002	3.78 × 10^−8^	1	21,434,533	343,524	30.26
rs172305	G	A	0.687	0.014	0.003	3.91 × 10^−8^	5	107,316,915	343,524	30.19
rs62618693	C	T	0.955	0.031	0.006	3.94 × 10^−8^	11	32,956,492	343,524	30.18
rs2011689	A	G	0.389	0.013	0.002	4.06 × 10^−8^	8	64,341,554	343,524	30.12
rs746839	G	C	0.377	0.014	0.003	4.44 × 10^−8^	8	142,617,261	343,524	29.95
rs7485554	A	G	0.572	0.014	0.002	4.63 × 10^−8^	12	84,070,366	343,524	29.87
rs62129471	A	G	0.658	0.014	0.003	4.87 × 10^−8^	19	1,950,864	343,524	29.77

Abbreviations: rs ID—SNP; effect allele—EA; other allele—OA; effect allele frequency—EAF; effect—beta; standard error—SE; *p*-value—*p*; chromosome—CHR; position—POS; F-statistics calculated using Bowden’s method—Fstats.

**Table 2:** Sensitivity analyses of the two-sample Mendelian randomization study between C-reactive protein and CVD outcomes using Approach 1.

CVD Outcomes	Intercept (SE)	Pleiotropy *p*	Cochran’s Q	Q_df	Het *p*
Myocardial Infarction	0.005 (0.003)	0.055	295.11	176	4.67 × 10^−8^
Coronary Artery Disease	0.006 (0.002)	0.005	500.58	169	1.54 × 10^−34^
Heart Failure	0.005 (0.002)	0.024	241.40	176	7.81 × 10^−4^
Atherosclerosis	0.004 (0.003)	0.253	283.14	176	5.37 × 10^−7^
Hypertensive Heart Disease	0.003 (0.004)	0.404	225.15	176	0.007

Abbreviations: number of SNPs in the outcome—CRP-associated SNPs; MR-Egger intercept (standard error)—Intercept (SE); *p*-value for the MR-Egger intercept—Pleiotropy *p*; *p*-value for the heterogeneity tests performed using the MR-Egger method—Het *p*.

**Table 3:** Results from the MR-PRESSO test between C-reactive protein and CVD outcomes using Approach 1.

CVD Outcomes	Raw	Raw *p*	Corrected	Corrected *p*
Myocardial Infarction	0.99 (0.90–1.09)	0.867	1.01 (0.92–1.10)	0.888
Coronary Artery Disease	0.99 (0.91–1.07)	0.750	1.04 (0.96–1.13)	0.316
Heart Failure	1.09 (1.01–1.18)	0.024	1.09 (1.01–1.17)	0.028
Atherosclerosis	1.17 (1.04–1.30)	0.006	1.15 (1.03–1.27)	0.011
Hypertensive Heart Disease	1.21 (1.06–1.37)	0.004	1.20 (1.06–1.36)	0.005

Abbreviations: estimated OR (95% confidence interval) for the raw MR analysis—Raw and the corresponding *p*-value—Raw *p*; estimated OR (95% confidence interval) for the outlier-corrected MR analysis—Corrected and the corresponding *p*-value—Corrected *p*.

**Table 4:** Sensitivity analyses of the two-sample Mendelian randomization study between C-reactive protein and CVD outcomes using Approach 2.

CVD Outcomes	Intercept (SE)	Pleiotropy *p*	Cochran’s Q	Q_df	Het *p*
Myocardial Infarction	0.004 (0.003)	0.199	196.59	134	3.49 × 10^−4^
Coronary Artery Disease	0.001 (0.002)	0.614	261.53	131	1.01 × 10^−10^
Heart Failure	0.007 (0.003)	0.010	177.88	134	0.007
Atherosclerosis	0.006 (0.004)	0.068	180.76	134	0.004
Hypertensive Heart Disease	0.007 (0.004)	0.123	161.67	134	0.052

Abbreviations: number of SNPs in the outcome—CRP-associated SNPs; MR-Egger intercept (standard error)—Intercept (SE); *p*-value for the MR-Egger intercept—Pleiotropy *p*; *p*-value for the heterogeneity tests performed using the MR-Egger method—Het *p*.

**Table 5:** Results from the MR-PRESSO test between C-reactive protein and CVD outcomes using Approach 2.

CVD Outcomes	Raw	Raw *p*	Corrected	Corrected *p*
Myocardial Infarction	1.01 (0.90–1.14)	0.873	1.03 (0.91–1.15)	0.656
Coronary Artery Disease	1.01 (0.93–1.10)	0.772	1.00 (0.92–1.09)	0.961
Heart Failure	1.05 (0.95–1.17)	0.343	NA	NA
Atherosclerosis	1.10 (0.96–1.27)	0.167	NA	NA
Hypertensive Heart Disease	1.16 (0.98–1.37)	0.092	NA	NA

Abbreviations: Estimated OR (95% confidence interval) for the raw MR analysis—Raw and the corresponding *p*-value—Raw *p*; estimated OR (95% confidence interval) for the outlier-corrected MR analysis—Corrected and the corresponding *p*-value—Corrected *p*. If the global test is not significant for the specified significance threshold (SignifThreshold = 0.05), no outliers can be detected, and the outlier-corrected analysis is NA.

**Table 6:** Results from the reverse causation inverse variance weighted two-sample MR analyses.

Exposure	Odds Ratio [Low 95% CI-High 95% CI]	*p*	# SNPs	Intercept (SE)	Pleiotropy *p*	Cochran’s Q	Q_df	Het *p*
Myocardial Infarction	0.93 [0.83–1.05]	0.248	7	0.011 (0.035)	0.766	420.82	5	9.65 × 10^−89^
Coronary Artery Disease	0.95 [0.84–1.08]	0.417	38	0.006 (0.015)	0.696	6080.02	36	0.000
Heart Failure	1.03 [0.98–1.07]	0.211	2	NA	NA	0.47	1	0.495
Atherosclerosis	1.09 [1.05–1.13]	2.16 × 10^−5^	1	NA	NA	NA	NA	NA
Hypertensive Heart Disease	0.99 [0.96–1.03]	0.728	2	NA	NA	2.46	1	0.117

Abbreviations: number of SNPs found in the outcome GWAS—# SNPs; MR-Egger intercept (standard error)—Intercept (SE); *p*-value for the MR-Egger intercept—Pleiotropy *p*; *p*-value for the heterogeneity tests performed using the MR-Egger method—Het *p*.

## Data Availability

The summary statistics used in this study can be obtained from http://app.mrbase.org/ (accessed on 9 January 2023).

## Supplementary Materials

The following supporting information can be downloaded at: https://www.mdpi.com/article/10.3390/ijms24119129/s1.

## Abbreviations

Cardiovascular disease: CVD, C-reactive protein: CRP, myocardial infarction: MI, coronary artery disease: CAD, heart failure: HF, hypertensive heart diseases: HHD, two-sample Mendelian randomization: MR, instrumental variables: IVs, multi-variable Mendelian randomization: MVMR, odds ratio: OR, 95% confidence interval: 95% CI, inverse-variance weighted: IVW, weighted median: WM, penalized weighted median: PWM, type 2 Diabetes: T2D, body mass index: BMI, type 2 diabetes adjusted for body mass index: T2DadjBMI, renal failure: RF, chronic kidney disease: CKD, UK Biobank: UKBB, pleiotropy *p*-value: Pleiotropy *p*, heterogeneity *p*-value: Het *p*.
