# Supplementary material for: C-Reactive Protein Levels and Risk of Cardiovascular Diseases: A Two-Sample Bidirectional Mendelian Randomization Study
Source: Int J Mol Sci. 2023 May 23;24(11):9129. doi: 10.3390/ijms24119129 (PMC10252732; doi:10.3390/ijms24119129)

**Supplementary Figures**

**Supplementary Figure S1:** Scatter plots of the causal association between CRP and CVD using Approach-1, (A) CRP-MI, (B) CRP-CAD, (C) CRP-HF, (D) CRP-Atherosclerosis, and (E) CRP-HHD MR study. The two-sample MR analyses were conducted using the MR-Egger, Inverse variance weighted, weighted median, and penalized weighted median methods. The estimated MR effect per method is depicted by the slope of each line.


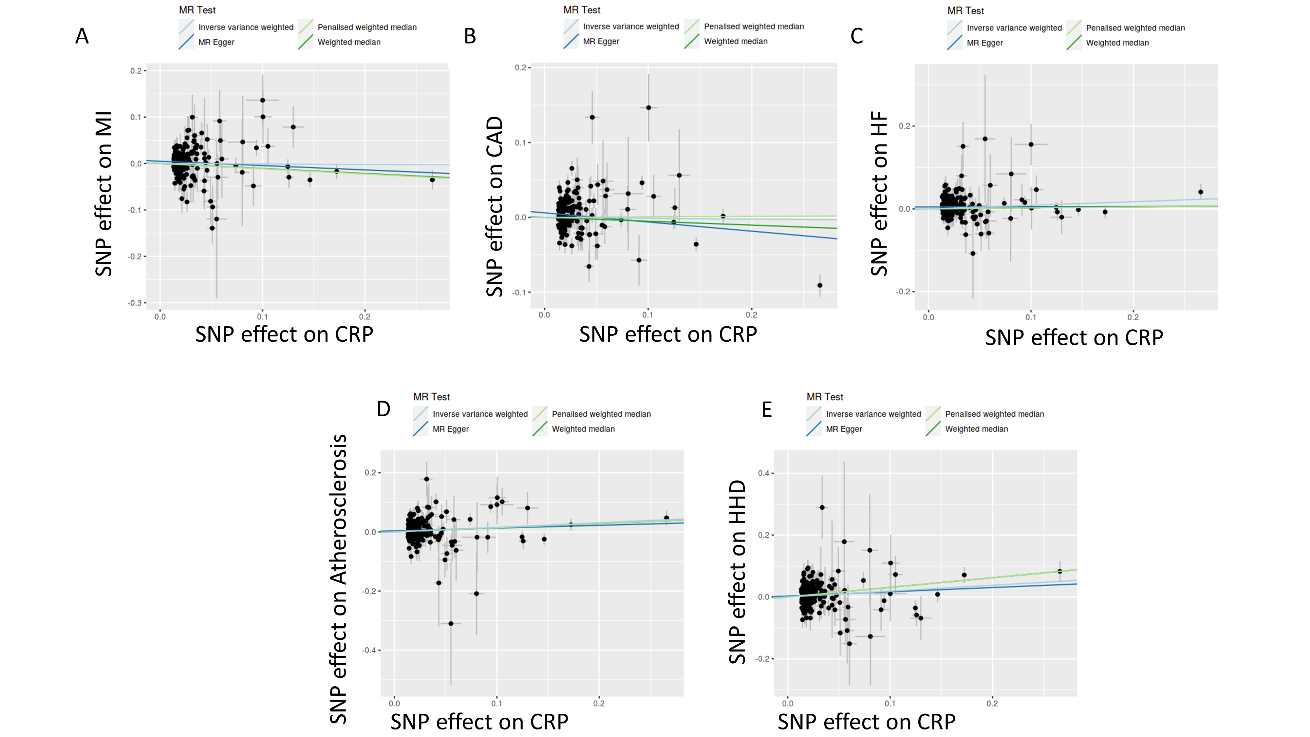


**Supplementary Figure S2:** Funnel plots of the causal association between CRP and CVD using Approach-1, (A) CRP-MI, (B) CRP-CAD, (C) CRP-HF, (D) CRP-Atherosclerosis, and (E) CRP-HHD MR study.


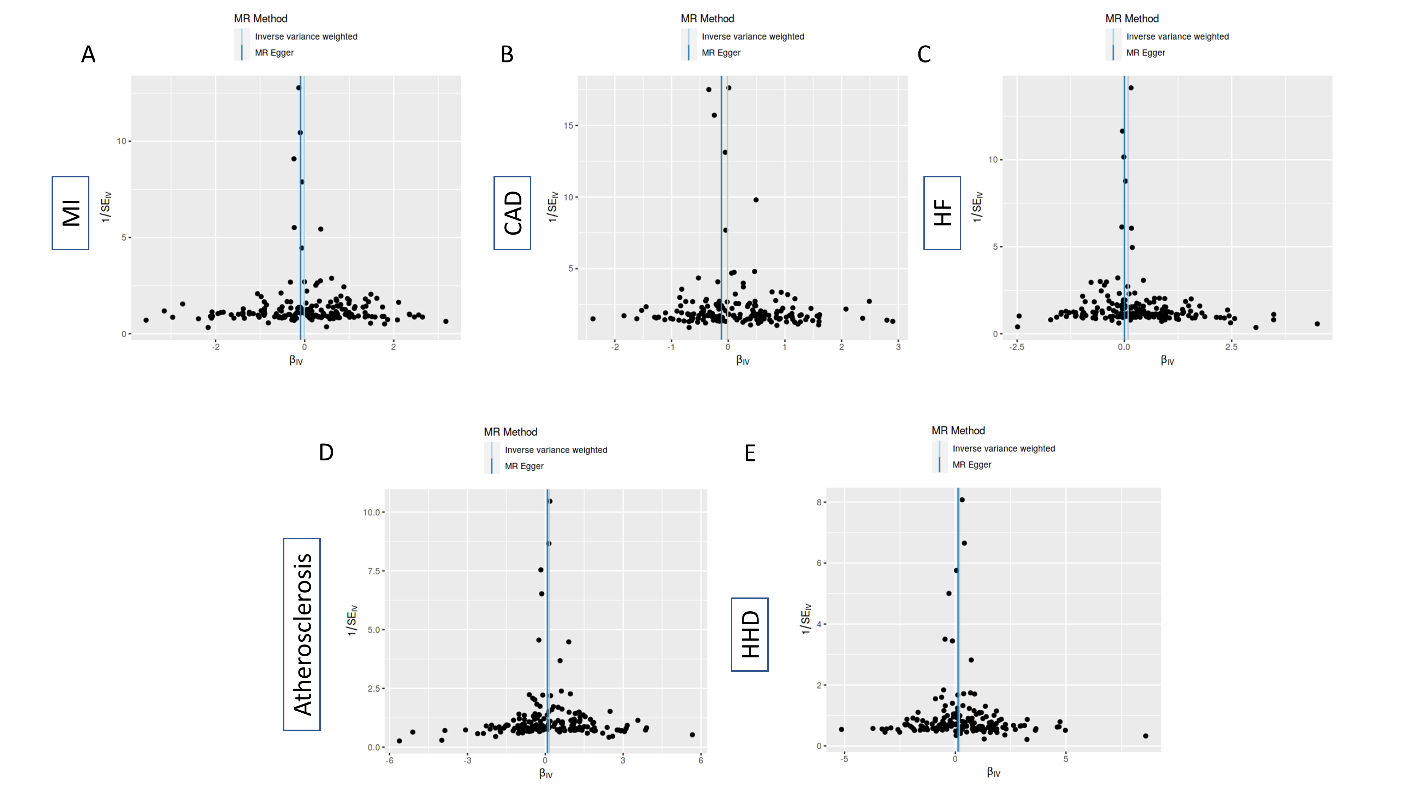


**Supplementary Figure S3:** Scatter plots of the causal association between CRP and CVD using Approach-2, (A) CRP-MI, (B) CRP-CAD, (C) CRP-HF, (D) CRP-Atherosclerosis, and (E) CRP-HHD MR study. The two-sample MR analyses were conducted using the MR-Egger, Inverse variance weighted, weighted median, and penalized weighted median methods. The estimated MR effect per method is depicted by the slope of each line.


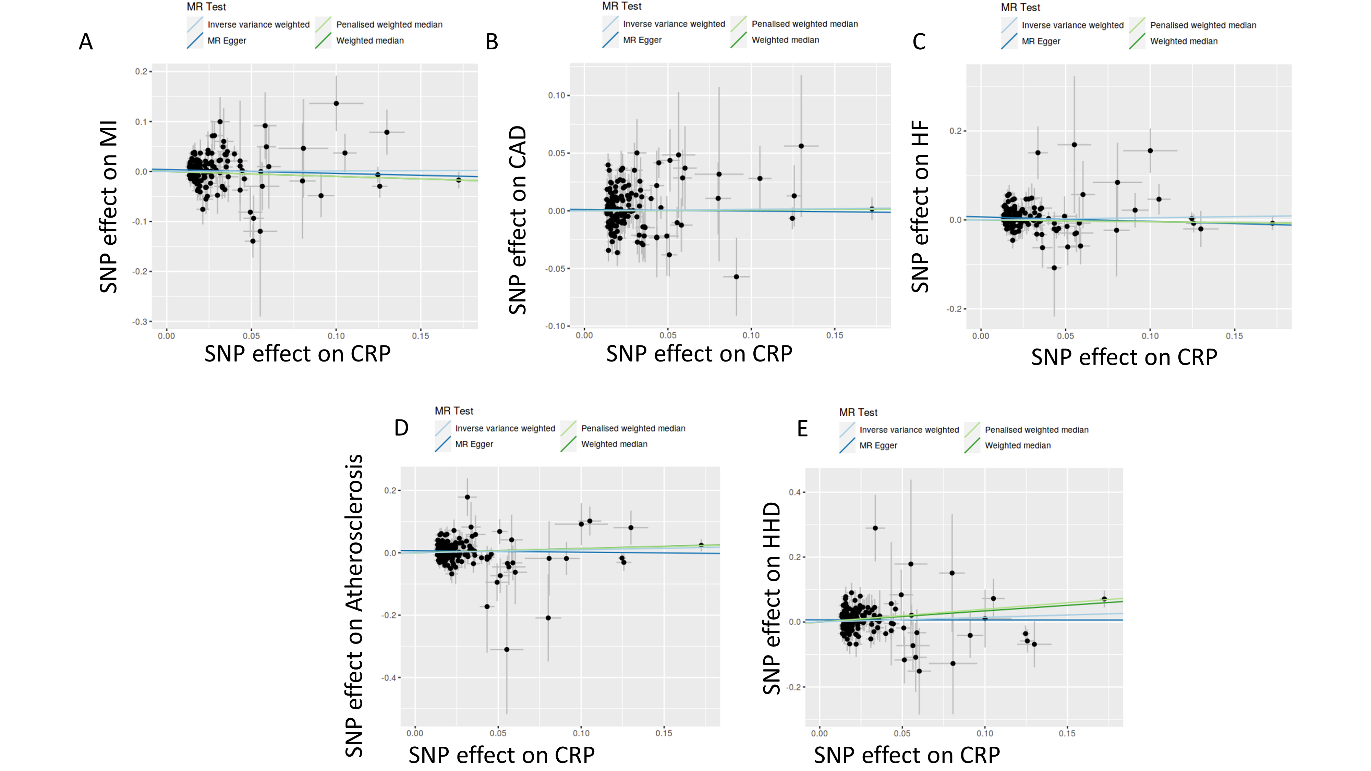


**Supplementary Figure S4:** Funnel plots of the causal association between CRP and CVD using Approach-2, (A) CRP-MI, (B) CRP-CAD, (C) CRP-HF, (D) CRP-Atherosclerosis, and (E) CRP-HHD MR study.


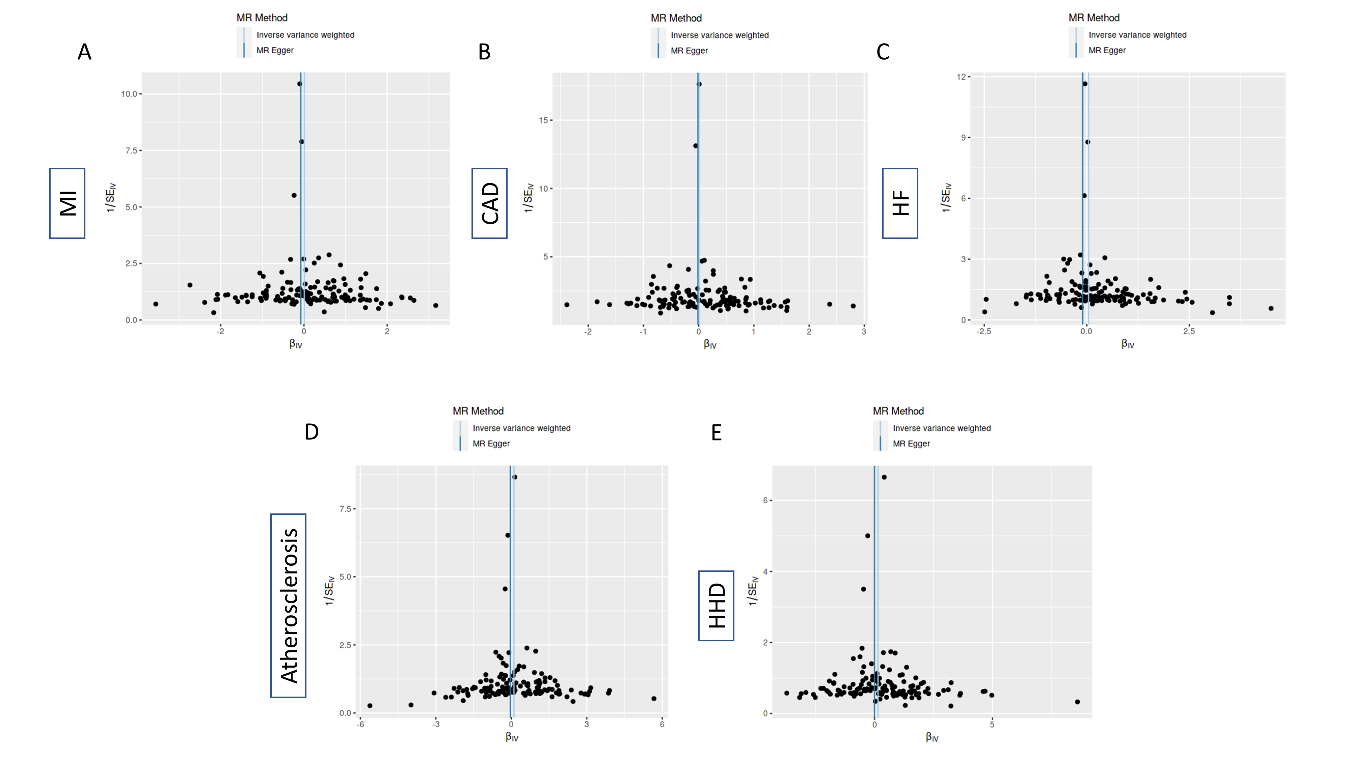

Supplement: Supplementary file 1 [file ijms-24-09129-s001.zip › ijms-2194936-supplementary.docx]
